# Supplementary material for: Dpb4 promotes resection of DNA double-strand breaks and checkpoint activation by acting in two different protein complexes
Source: Nat Commun. 2021 Aug 6;12:4750. doi: 10.1038/s41467-021-25090-9 (PMC8346560; doi:10.1038/s41467-021-25090-9)
Supplement: Supplementary file 1 — Supplementary Information [file 41467_2021_25090_MOESM1_ESM.pdf]

Supplementary Information

Dpb4 promotes resection of DNA double-strand breaks and checkpoint activation by acting in two different protein complexes

Erika Casari, Elisa Gobbini, Marco Gnugnoli, Marco Mangiagalli, Michela Clerici and Maria Pia Longhese

Content:

Supplementary Figures 1-6

Supplementary Tables 1-3

Supplementary Figure 1

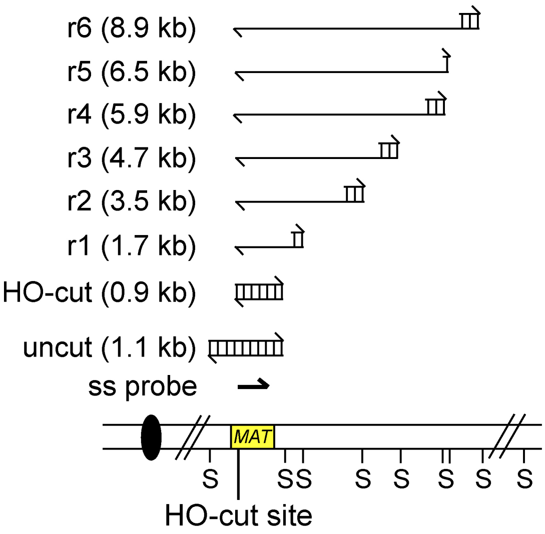

Supplementary Fig. 1. System used to detect DSB resection. Resection of the DSB end progressively eliminates SspI sites (S), producing longer SspI fragments (r1 through r6) that can be separated on an alkaline agarose gel and visualized after hybridization with a single-stranded RNA probe that anneals to the unresected strand at one side of the DSB.

Supplementary Figure 2

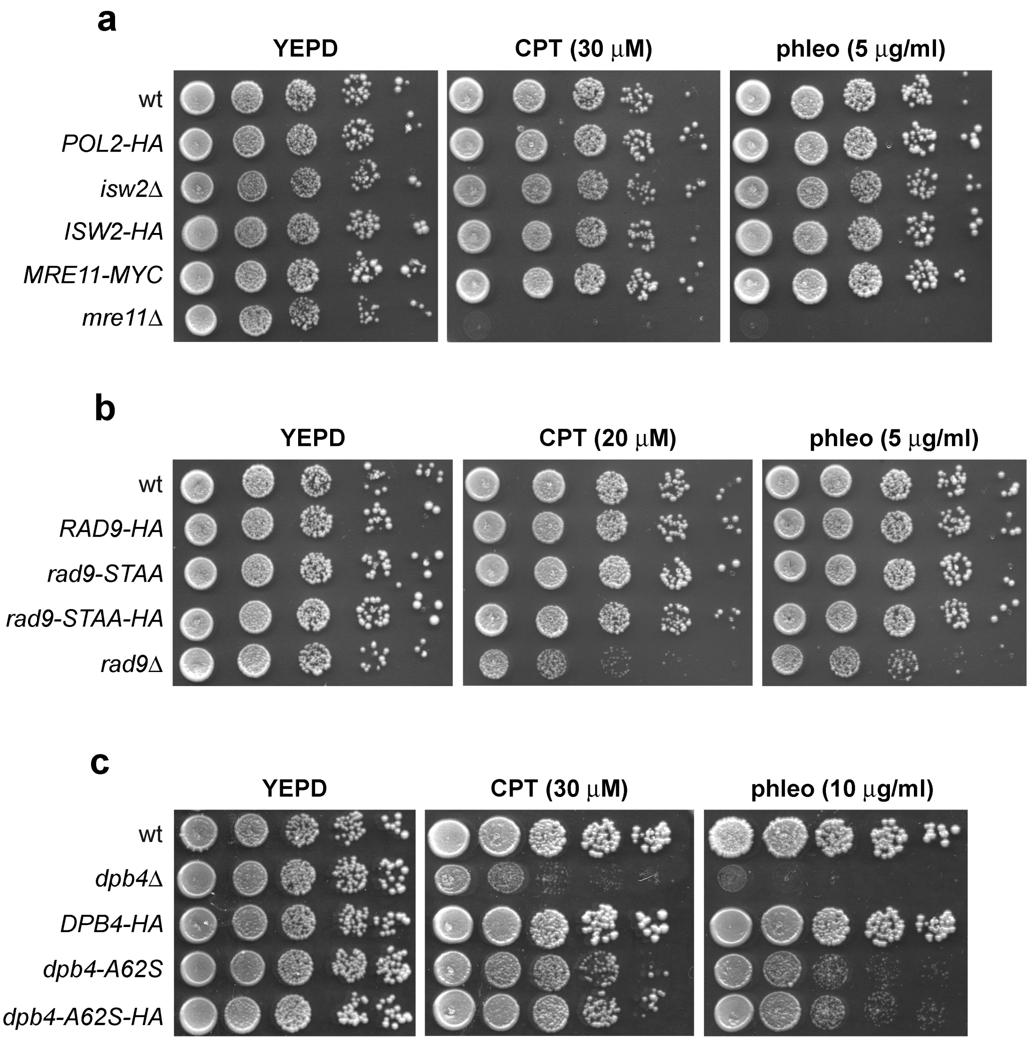

Supplementary Fig. 2. DNA damage sensitivity of cells expressing HA- or Myc-tagged proteins.
a-c Exponentially growing cell cultures were serially diluted (1:10) and each dilution was spotted out onto YEPD plates with or without camptothecin (CPT) or phleomycin (phleo) at the indicated concentrations.

Supplementary Figure 3

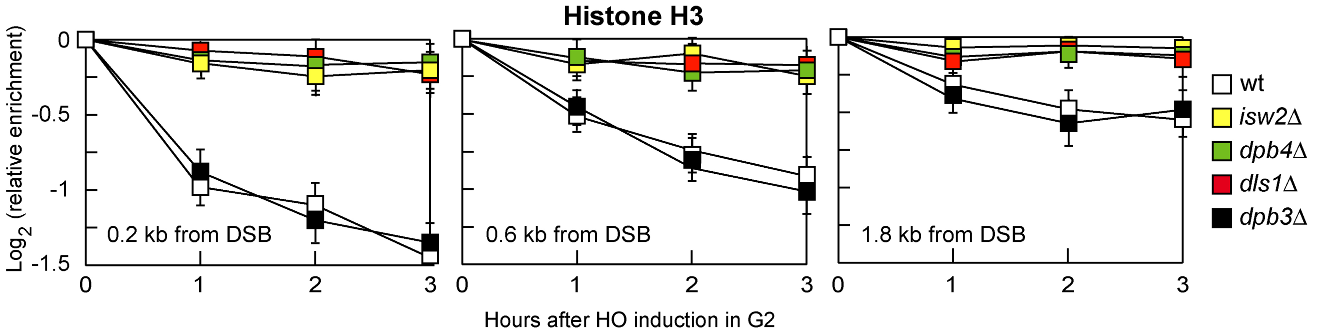

Supplementary Fig. 3. The lack of Dls1 or Isw2, but not of Dpb3, impairs histone H3 removal from DSBs. Exponentially growing YEPR cell cultures of JKM139 derivative strains were transferred to YEPRG to induce HO expression. Relative fold enrichment of histone H3 at the HO-induced DSB was evaluated after ChIP with anti-H3 antibody and qPCR . The mean values of three independent experiments are represented with error bars denoting s.d. See source data file for the statistical analysis that was performed using unpaired two-tailed Student's *t*-test.

Supplementary Figure 4

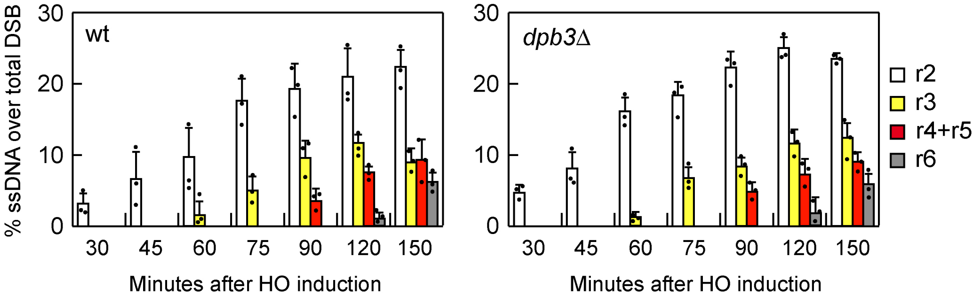

Supplementary Fig. 4. Densitometric analysis of the resection products of Figure 7a. The mean values of three independent experiments are represented with error bars denoting s.d. See source data file for the statistical analysis that was performed using unpaired two-tailed Student's *t*-test.

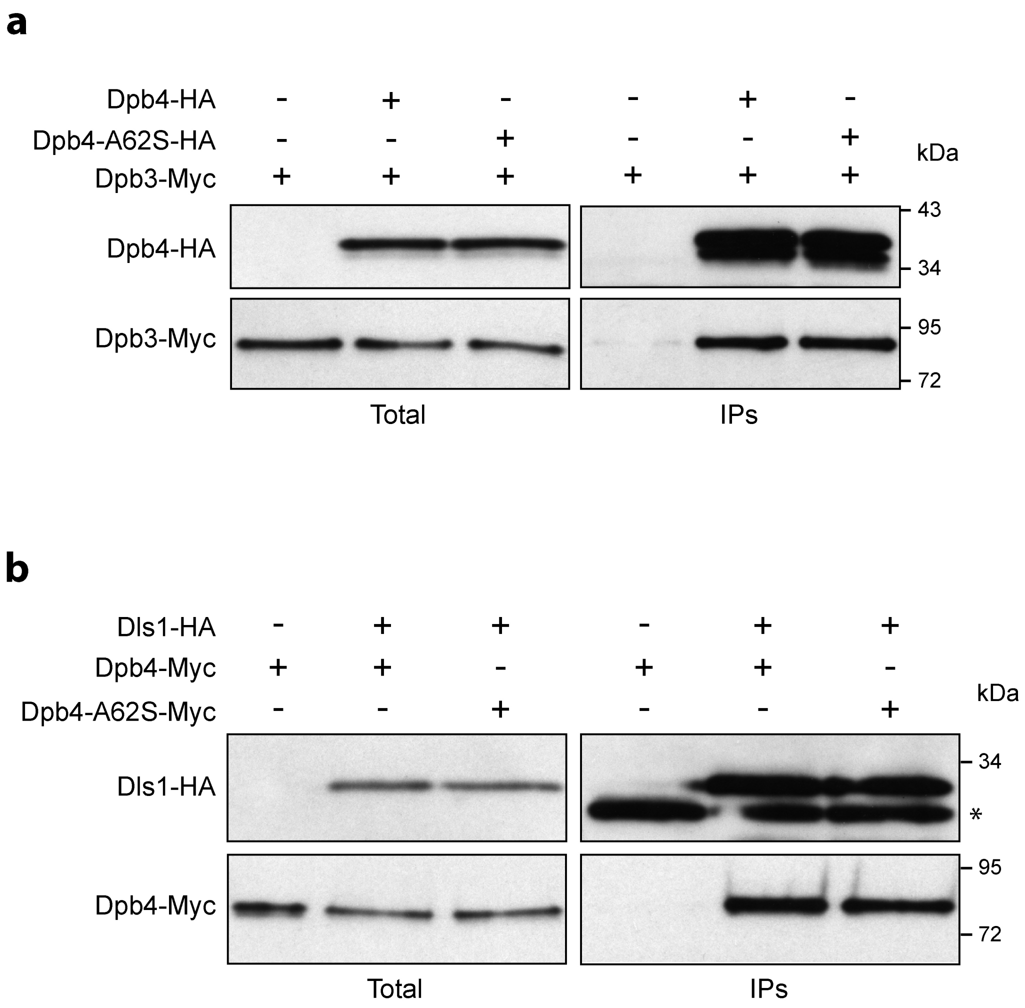

Supplementary Fig. 5. The A62S mutation does not affect Dpb3-Dpb4 and Dls1-Dpb4 complex formation. a, b Protein extracts from exponentially growing cells treated with phleomycin (15 µg/ml) were analyzed by western blotting with anti-HA or anti-Myc antibodies either directly (total) or after immunoprecipitation (IP) with anti-HA antibody. The asterisk indicates the immunoglobulins present in the immunoprecipitates. The experiments in (**a**) and (**b**) were performed independently two times with similar results.

Supplementary Figure 6

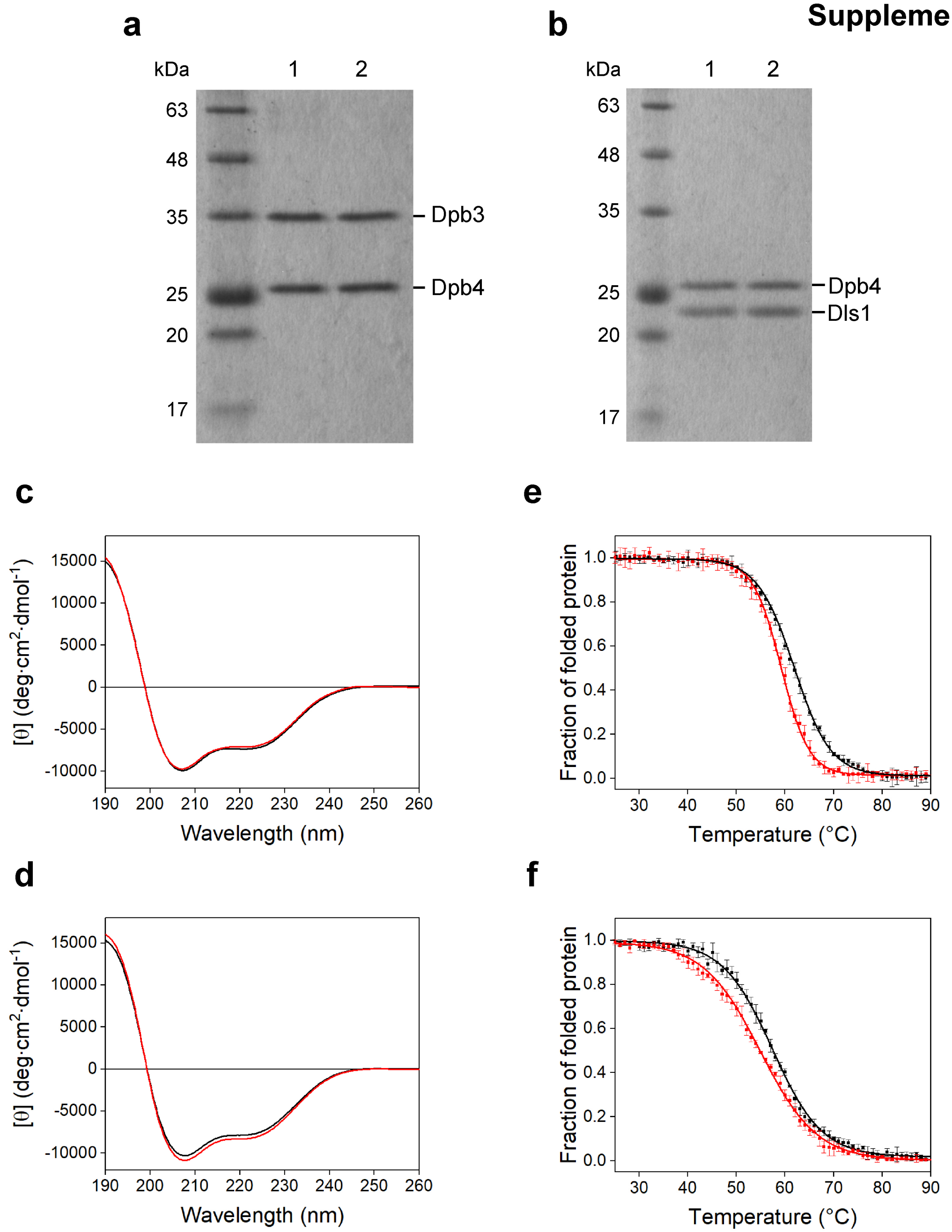

Supplementary Fig. 6. Purification of Dpb3-Dpb4 and Dls1-Dpb4 heterodimers. a SDS-PAGE analysis of purified Dpb3-Dpb4 (lane 1) and Dpb3-Dpb4^{A62S} (lane 2) complexes. **b** SDS-PAGE analysis of purified Dls1-Dpb4 (lane 1) and Dls1-Dpb4^{A62S} (lane 2) complexes. The experiments in (**a**) and (**b**) were performed independently two times with similar results. **c** Far-UV CD spectra of Dpb3-Dpb4 (black line) and Dpb3-Dpb4^{A62S} (red line). **d** Far-UV CD spectra of Dls1-Dpb4 (black line) and Dls1-Dpb4^{A62S} (red line). For (**c**) and (**d**) one of three independent measurements was shown. **e** Thermal stability of Dpb3-Dpb4 (black line) and Dpb3-Dpb4^{A62S} (red line). The mean values of three independent measurements are represented with error bars indicating s.d. **f** Thermal stability of Dls1-Dpb4 (black line) and Dls1-Dpb4^{A62S} (red line). The mean values of three independent measurements are represented with error bars indicating s.d.

Supplementary Table 1. ***Saccharomyces cerevisiae*** **strains used in this study.**

**Strain**	**Relevant genotype**	**Source**
W303	*MAT*a/α *ade2-1 can1-100 his3-11,15 leu2-3,112 trp1-1 ura3-1 rad5-535*	
YLL 1069.3	W303 *MAT*a *sae2Δ::KANMX*	1
DMP 3335/2A	W303 *MAT*a *tel1Δ::HIS3*	1
DMP 6389/2D	W303 *MAT*α *dpb4-A62S::KANMX*	This study
DMP 6389/3C	W303 *MAT*a *dpb4-A62S::KANMX*	This study
YLL 3759.1	W303 *MAT*α *dpb4Δ::HPHMX*	This study
DMP 7039/3D	W303 *MAT*a *dpb4-A62S::KANMX sae2Δ::HIS3*	This study
DMP 7039/7C	W303 *MAT*a *dpb4-A62S::KANMX sae2Δ::HIS3*	This study
DMP 7039/5C	W303 *MAT*a *dpb4Δ::HPHMX sae2Δ::HIS3*	This study
DMP 7039/8D	W303 *MAT*a *dpb4Δ::HPHMX sae2Δ::HIS3*	This study
DMP 7040/10C	W303 *MAT*α *dpb4-A62S::KANMX tel1Δ::HIS3*	This study
DMP 7040/1A	W303 *MAT*a *dpb4-A62S::KANMX tel1Δ::HIS3*	This study
DMP 7040/8D	W303 *MAT*α *dpb4Δ::HPHMX tel1Δ::HIS3*	This study
DMP 7040/4C	W303 *MAT*a *dpb4Δ::HPHMX tel1Δ::HIS3*	This study
DMP 2949/1C	W303 *MAT*a *rad9Δ::URA3*	2
DMP 7064/10A	W303 *MAT*a *rad9Δ::URA3 tel1Δ::HIS3*	This study
DMP 7065/11D	W303 *MAT*a *rad9Δ::URA3 dpb4-A62S::KANMX*	This study
DMP 7066/17A	W303 *MAT*a *rad9Δ::URA3 dpb4-A62S::KANMX tel1Δ::HIS3*	This study
DMP 7196/5C	W303 *MAT*a *rad9Δ::URA3 sae2Δ::HIS3*	This study
DMP 7197/10A	W303 *MAT*a *rad9Δ::URA3 dpb4-A62S:: KANMX sae2Δ::HIS3*	This study
DMP 2760/3B	W303 *MAT*a *rad53-K227A::KANMX*	3
DMP 7226/2C	W303 *MAT*α *rad53-K227A::KANMX dpb4-A62S::KANMX*	This study
DMP 7225/9B	W303 *MAT*a *rad53-K227A::KANMX sae2Δ::HIS3*	This study
DMP 7225/5B	W303 *MAT*a *rad53-K227A::KANMX dpb4-A62S::KANMX sae2Δ::HIS3*	This study
YLL 4189.3	W303 *MAT*a *isw2Δ::HIS3*	This study
DMP 7047/4D	W303 *MAT*α *tel1Δ::HIS3 isw2Δ::HIS3*	This study
DMP 7115/2D	W303 *MAT*a *isw2Δ::HIS3 dpb4-A62S::KANMX*	This study
DMP 7116/9B	W303 *MAT*a *tel1Δ::HIS3 isw2Δ::HIS3 dpb4-A62S::KANMX*	This study
YLL 4277.1	W303 *MAT*a *dls1Δ::URA3*	This study
DMP 7143/1D	W303 *MAT*a *dls1Δ::URA3 tel1Δ::HIS3*	This study

DMP 7152/7B	W303 *MAT*a *dls1Δ::URA3 dpb4-A62S::KANMX*	This study
DMP 7153	W303 *MAT*a *dls1Δ::URA3 tel1Δ::HIS3 dpb4-A62S::KANMX*	This study
DMP 4302/3	W303 *MAT*a *dpb3Δ::URA3*	This study
YLL 4191.1	W303 *MAT*α *DPB4-18MYC::URA3*	This study
DMP 7350/6A	W303 *MAT*a *dpb4-A62S-3HA::TRP1 DPB3-18MYC::URA3*	This study
DMP 7351/1C	W303 *MAT*a *DPB4-3HA::TRP1 DPB3-18MYC::URA3*	This study
DMP 7355/2C	W303 *MAT*a *DLS1-3HA::TRP1 DPB4-18MYC::URA3*	This study
YLL 4190	W303 *MAT*α *DPB3-18MYC::URA3*	This study
DMP 7356/10B	W303 *MAT*a *DLS1-3HA::TRP1 dpb4-A62S-18MYC::URA3*	This study
JKM 139	*MATa hmlD::ADE1 hmrD::ADE1 ade1-100 lys5 leu2-3, 112 trp::hisG ura3-52 ho ade3::GAL-HO site*	4
YLL 4182.1	JKM139 *MAT*a *dpb4Δ::HPHMX*	This study
YLL 4227.23	JKM139 *MAT*a *dpb4-A62S::KANMX*	This study
YLL 1523.3	JKM139 *MAT*a *sae2Δ::KANMX*	1
YLL 1794.3	JKM139 *MAT*a *tel1Δ::NATMX*	1
DMP 7069/18A	JKM139 *MAT*a *dpb4-A62S::KANMX tel1Δ::NATMX*	This study
DMP 7070/14B	JKM139 *MAT*a *dpb4-A62S::KANMX sae2Δ::HPHMX*	This study
YLL 4311/A	JKM139 *MAT*a *dpb4-A62S-3HA::TRP1*	This study
YLL 4312.3	JKM139 *MAT*a *DPB4-3HA::TRP1*	This study
YLL 4344.A	JKM139 *MAT*a *dpb3Δ::URA3*	This study
YLL 4264.1	JKM139 *MAT*a *isw2Δ::HPHMX*	This study
YLL 4276.8	JKM139 *MAT*a *dls1Δ::URA3*	This study
YLL 1854.2	JKM139 *MAT*a *MRE11-18MYC::TRP1*	5
DMP 7281/1D	JKM139 *MAT*a *dpb4Δ::HPHMX MRE11-18MYC::TRP1*	This study
DMP 7137/11A	JKM139 *MAT*a *dpb4-A62S::KANMX MRE11-18MYC::TRP1*	This study
DMP 7195/7D	JKM139 *MAT*a *isw2Δ::HPHMX MRE11-18MYC::TRP1*	This study
DMP 7298/1D	JKM139 *MAT*a *dls1Δ::URA3 MRE11-18MYC::TRP1*	This study
DMP 7280/11D	JKM139 *MAT*a *dpb3Δ::URA3 MRE11-18MYC::TRP1*	This study
DMP 7323/3B	JKM139 *MAT*a *isw2Δ::HPHMX dpb4Δ::HPHMX MRE11-18MYC::TRP1*	This study
YLL 3421.2	JKM139 *MAT*a *RAD9-3HA::TRP1*	6
DMP 7107/1A	JKM139 *MAT*a *isw2Δ::HPHMX RAD9-3HA::TRP1*	This study
DMP 7071/6D	JKM139 *MAT*a *dpb4-A62S::KANMX RAD9-3HA::TRP1*	This study
DMP 7101/1D	JKM139 *MAT*a *dpb4Δ::HPHMX RAD9-3HA::TRP1*	This study

DMP 7246/4D	JKM139 *MAT*a *dpb3Δ::URA3 RAD9-3HA::TRP1*	This study
DMP 7324/5D	JKM139 *MAT*a *dpb3Δ::URA3 dpb4Δ::HPHMX RAD9-3HA::TRP1*	This study
DMP 7244/4B	JKM139 *MAT*a *ddc1-T602A::URA3 DPB4-3HA::TRP1*	This study
DMP 7245/2A	JKM139 *MAT*a *ddc1Δ::KANMX DPB4-3HA::TRP1*	This study
DMP 7271/9C	JKM139 *MAT*a *rad24Δ::KANMX DPB4-3HA::TRP1*	This study
YLL 4345.9	JKM139 *MAT*a *rad9Δ::KANMX TRP1::rad9-ST462,474AA-3HA::URA3*	This study
DMP 7285/10B	JKM139 *MAT*a *dpb4Δ::HPHMX rad9Δ::KANMX TRP1::rad9-ST462,474AA-3HA::URA3*	This study
DMP 7317/7A	JKM139 *MAT*a *RAD9-3HA::TRP1 dot1Δ::KANMX bar1Δ::TRP1 dpb4Δ::HPHMX*	This study
DMP 7317/7D	JKM139 *MAT*a *RAD9-3HA::TRP1 bar1Δ::TRP1*	This study
DMP 7317/9A	JKM139 *MAT*a *RAD9-3HA::TRP1 dot1Δ::KANMX bar1Δ::TRP1*	This study
DMP 7317/9D	JKM139 *MAT*a *RAD9-3HA::TRP1 bar1Δ::TRP1 dpb4Δ::HPHMX*	This study
DMP 7328/10D	JKM139 *MAT*a *dpb4Δ::HPH RAD9-3HA::TRP1 hta2Δ::NATMX hta1-S129A::URA3*	This study
YLL 3426.1	JKM139 *MAT*a *RAD9-3HA::TRP1 hta2Δ::NATMX hta1-S129A::URA3*	This study
DMP 7348/5C	JKM139 *MAT*a *ISW2-3HA::TRP1 dpb4-A62S::KANMX*	This study
DMP 7349/3A	JKM139 *MAT*a *ISW2-3HA::TRP1 dpb4Δ::HPHMX*	This study
YLL 4382.14	JKM139 *MAT*a *dpb4-A62S::KANMX POL2-3HA::URA3*	This study
DMP 7354/3C	JKM139 *MAT*a *dpb4Δ::HPHMX POL2-3HA::URA3*	This study
YLL 4382.57	JKM139 *MAT*a *POL2-3HA::URA3*	This study
DMP 5793/6C	JKM139 *MAT*a *rad9Δ::KANMX*	This study
YLL1769.1	JKM139 *MAT*a *mre11Δ::NATMX*	1
YLL 4274.9	JKM139 *MAT*a *ISW2-3HA::TRP1*	This study
YLL 4176.17	JKM139 *MAT*a *rad9Δ::KANMX TRP1::rad9-ST462,474AA::URA3*	This study

Supplementary Table 2. Oligonucleotides used for gene tagging and disruptions.

Name	Sequence (5'-3')
PRP1589	GCCAAGTATTACGGTTTTGATATATTATTGAGTTGTATTGCTGATTTGAC CATATCCGGTCTGCTGCTAG
PRP1590	TTTTTCATGGTAAAGAGGCCATTGAACCTCGCGTTATATACTGCTTACT CACCTCGAGGCCAGAAGAC
PRP1989	CAGACCATATATTTTTACACACGATGCCACCAAAAGGTTGGAGCGTAC GCTGCAGGTCGAC
PRP1990	TGGGAGTGGTGGCAAGCACTACTAGACAGTTTCCATAATCGATGAATT CGAGCTCG
PRP2007	CGTTAACAAGAAATTATGGCGTTGAGGAAGTGTATTAGCGCGTACGCT GCAGGTCGAC
PRP2008	CACAAAGTGGCTCTAACTAAACCTGCTTGATATCGTATCTATCGATGAA TTCGAGCTCG
PRP2316	GAGCCAAGATGTAGAAACTAGAGTTCAAAACCTTGAGCAAACGTCCGG TTCTGCTGCTAG
PRP2317	TGGGAGTGGTGGCAAGCACTACTAGACAGTTTCCATAGCGGGGCCTCG AGGCCAGAAGAC
PRP2320	AGGAAAACTTACAATCAGATCATGACGACCCAGCAAGAGGACGTACGC TGCAGGTCGAC
PRP2321	CTCTCACGTCACTTATTTTAATGCACAATACATGATTCATGCATCGATG AATTCGAGCTCG
PRP2328	TCTGATATCGAAGTTGACCATACGAAAAGCACCGATCCTTCCGGTTCTG CTGCTAG
PRP2329	GCATCGAATAGTAATTACATAGCAATAATAGCAACAACACCTCGAGGC CAGAAGAC
PRP2414	CGATCATGTTGATAAAAGAACCAAAATTGATCAAGAAGCATCCGGTTC TGCTGCTAG
PRP2415	TTATATCTCTCACGTCACTTATTTTAATGCACAATACATGATCCTCGAG GCCAGAAGAC
PRP2425	GATGTGATCAGGCTAACACAATGAACAACGAGACTAGTGGTGAAAGAG AGAGAGAGAAGC
PRP2426	CGTGAGAGAGAGAAGAAAAATCCTTCTATGCAATGTGCTTGTGGTCGT TCATGGTGACACTTT
PRP2446	GCAAGGGTCAACCGTGTTGCAAAAAAAATGTCCAACTTAGTTAAAGAT GAAAGAGAGAGAGAGAAGC
PRP2447	CGAATAGTAATTACATAGCAATAATAGCAACAACACTAAGGATCGGTG GTCGTTCATGGTGACACTTT
PRP2565	TAATGACGAAGACGACGACGAAGACGTTACTGATCAAGAATCCGGTTC TGCTGCTAG
PRP2566	GAGAGAGAGAAGAAAAATCCTTCTATGCAATGTGCTTGTGTAGCCTCG AGGCCAGAAGAC

Supplementary Table 3. Oligonucleotides used for qPCR.

**Name**	**Sequence (5'-3')**
ARO+	TGAGTCGTTACAAGGTGATGCC
ARO-	ACCTACAGGAGGACCCGAAA
DSB 0.2+	TCAGACTCAAGCAAACAATCAA
DSB 0.2-	CCCGTATAGCCAATTCGTTC
DSB 0.6+	CACCCAAGAAGGCGAATAAG
DSB 0.6-	CATGCGGTTCACATGACTTT
DSB 1.8+	ACGTCGTTGTTAATGGTGGTG
DSB 1.8-	CGCGAGTCTTATGCCAAAAA

References

1. Gobbini, E., Villa, M., Gnugnoli, M., Menin, L., Clerici, M. & Longhese, M. P. Sae2 function at DNA double-strand breaks is bypassed by dampening Tel1 or Rad53 activity. *PLoS Genet.* **11**, e1005685 (2015).
2. Bonetti, D., Villa, M., Gobbini, E., Cassani, C., Tedeschi, G. & Longhese, M. P. Escape of Sgs1 from Rad9 inhibition reduces the requirement for Sae2 and functional MRX in DNA end resection. *EMBO Rep.* **16,** 351-361 (2015).
3. Longhese, M. P., Paciotti, V., Neecke, H. & Lucchini, G. Checkpoint proteins influence telomeric silencing and length maintenance in budding yeast. *Genetics* **155**, 1577-1591 (2000).
4. Lee, S. E., Moore, J. K., Holmes, A., Umezu, K., Kolodner, R.D. & Haber, J. E. *Saccharomyces* Ku70, Mre11/Rad50 and RPA proteins regulate adaptation to G2/M arrest after DNA damage. *Cell* **94**, 399-409 (1998).
5. Clerici, M., Mantiero, D., Lucchini, G. & Longhese MP. The *Saccharomyces cerevisiae* Sae2 protein negatively regulates DNA damage checkpoint signalling. *EMBO Rep.* **7**, 212-218 (2006).
6. Clerici, M., Trovesi, C., Galbiati, A., Lucchini, G. & Longhese, M. P. Mec1/ATR regulates the generation of single-stranded DNA that attenuates Tel1/ATM signaling at DNA ends. *EMBO J.* **33**, 198-216 (2014).
