# Supplementary material for: Dpb4 promotes resection of DNA double-strand breaks and checkpoint activation by acting in two different protein complexes
Source: Nat Commun. 2021 Aug 6;12:4750. doi: 10.1038/s41467-021-25090-9 (PMC8346560; doi:10.1038/s41467-021-25090-9)
Supplement: Supplementary file 3 — Reporting Summary [file 41467_2021_25090_MOESM3_ESM.pdf]

nature research

Corresponding author(s): Maria Pia Longhese

Last updated by author(s): Jun 22, 2021

Reporting Summary

Nature Research wishes to improve the reproducibility of the work that we publish. This form provides structure for consistency and transparency in reporting. For further information on Nature Research policies, see our Editorial Policies and the Editorial Policy Checklist.

Statistics

For all statistical analyses, confirm that the following items are present in the figure legend, table legend, main text, or Methods section.

n/a	Confirmed	
☐	☒	The exact sample size (*n*) for each experimental group/condition, given as a discrete number and unit of measurement
☒	☐	A statement on whether measurements were taken from distinct samples or whether the same sample was measured repeatedly
☐	☒	The statistical test(s) used AND whether they are one- or two-sided<br>*Only common tests should be described solely by name; describe more complex techniques in the Methods section.*
☒	☐	A description of all covariates tested
☒	☐	A description of any assumptions or corrections, such as tests of normality and adjustment for multiple comparisons
☐	☒	A full description of the statistical parameters including central tendency (e.g. means) or other basic estimates (e.g. regression coefficient) AND variation (e.g. standard deviation) or associated estimates of uncertainty (e.g. confidence intervals)
☐	☒	For null hypothesis testing, the test statistic (e.g. *F*, *t*, *r*) with confidence intervals, effect sizes, degrees of freedom and *P* value noted<br>*Give P values as exact values whenever suitable.*
☒	☐	For Bayesian analysis, information on the choice of priors and Markov chain Monte Carlo settings
☒	☐	For hierarchical and complex designs, identification of the appropriate level for tests and full reporting of outcomes
☒	☐	Estimates of effect sizes (e.g. Cohen's *d*, Pearson's *r*), indicating how they were calculated

Our web collection on statistics for biologists contains articles on many of the points above.

Software and code

Policy information about availability of computer code

Data collection	Bio-Rad CFX Connect™ v. 1.1 (4.1.2433.1219) for quantitative PCR data
Data analysis	Microsoft Excel Professional 365<br>Scion Image Beta 4.0.2<br>Pymol 2.4.1<br>UCSF Chimera X 0.93<br>OriginPro 2020<br>Bio-Rad CFX Maestro 1.1

For manuscripts utilizing custom algorithms or software that are central to the research but not yet described in published literature, software must be made available to editors and reviewers. We strongly encourage code deposition in a community repository (e.g. GitHub). See the Nature Research guidelines for submitting code & software for further information.

Data

Policy information about availability of data

All manuscripts must include a data availability statement. This statement should provide the following information, where applicable:

- Accession codes, unique identifiers, or web links for publicly available datasets
- A list of figures that have associated raw data
- A description of any restrictions on data availability

All data are in the paper and supplementary information. The source data underlying Figs. 2a-e, 3a-d, 5a-d, 6a-f, 7a-d, g, h, 8c-f and Supplementary Figs. 3, 4, 5a, b, 6a-f are provided as a Source data file. Dpb3-Dpb4 structure was extracted from PDB 6WJV (http://doi.org/10.2210/pdb6WJV/pdb). All data are available from the authors upon reasonable request.

Field-specific reporting

Please select the one below that is the best fit for your research. If you are not sure, read the appropriate sections before making your selection.

☒ Life sciences ☐ Behavioural & social sciences ☐ Ecological, evolutionary & environmental sciences

For a reference copy of the document with all sections, see nature.com/documents/nr-reporting-summary-flat.pdf

Life sciences study design

All studies must disclose on these points even when the disclosure is negative.

Sample size	Sample size was not determined statistically, as this study did not include animal models or human participants. Sample size was always chosen to be large enough to minimize stochastic effects and ensures reproducibility of the data. We used 400 million cells for each ChIP sample, 400 million cells for each resection sample, 10 million cells for each western blot sample, 50,000 cells for the first drop in the viability assays.
Data exclusions	No data were excluded.
Replication	All the experiments were reliably reproduced and were obtained from 3 independent biological replicates, with the exception of Figure 3b that was performed independently four times, Figures 3a, 7b, c, 8e, f and Supplementary Figures 5a, b and 6a, b that were performed independently two times.
Randomization	Cell samples were always chosen randomly to avoid any possible bias. Each genotype or treatment was paired with the respective controls.
Blinding	Blinding was not performed in this study as data collection and analysis were objectively obtained/quantified and therefore not subjected to subjective judgment.

Reporting for specific materials, systems and methods

We require information from authors about some types of materials, experimental systems and methods used in many studies. Here, indicate whether each material, system or method listed is relevant to your study. If you are not sure if a list item applies to your research, read the appropriate section before selecting a response.

Materials & experimental systems

n/a	Involved in the study	
☐	☒	Antibodies
☒	☐	Eukaryotic cell lines
☒	☐	Palaeontology and archaeology
☒	☐	Animals and other organisms
☒	☐	Human research participants
☒	☐	Clinical data
☒	☐	Dual use research of concern

Methods

n/a	Involved in the study	
☒	☐	ChIP-seq
☒	☐	Flow cytometry
☒	☐	MRI-based neuroimaging

Antibodies

Antibodies used	Rabbit anti-Rad53 polyclonal antibody, Abcam ab104232 (RRID:AB_2687603)<br>Mouse Anti-HA (12CA5) monoclonal in-house antibody<br>Mouse Anti-Myc (9E10) monoclonal in-house antibody<br>Rabbit Anti-H2A polyclonal antibody, Active Motif 39945 (RRID:AB_2793402)<br>Rabbit Anti-H3 polyclonal antibody, Abcam ab1791 (RRID:AB_302613)
Validation	We have validated the specificity of the anti-HA and anti-MYC antibodies in Western Blot by using protein extracts from S. cerevisiae yeast strains without any tag.<br>Application (western blotting), species reactivity (S. cerevisiae) and validation (e.g. DOI: 10.1016/j.molcel.2018.11.025) for the anti-Rad53 antibody were indicated on the Abcam website.<br>Application (ChIP), species reactivity (S. cerevisiae) and validation (e.g. DOI: 10.1534/genetics.117.300359) for the anti-H3 antibody were indicated on the Abcam website.<br>Application (ChIP), species reactivity (S. cerevisiae) and validation for the anti-H2A antibody were indicated on the Active Motif website.
